# Infection

**Published:** 1881-02

**Authors:** 


### INFECTION.

The etiological connection between micro-organism and certain pathological or septic processes is still *sub judice.* Arguments, whether negative or affirmative, are very specious, and one's convictions will fluctuate according to the party he listens to. Hiller and Billroth advance very ingenious arguments against the connection. Hillroth directs attention to the fact that micro-cocci may abound in cadavers where death has not been caused by septic influences, and infers from this that their presence may be of secondary significance. He further concludes that bacteria, as parasites, are harmless, but that their absorption of septic molecules renders them hurtful. In this he considers the septic molecules as the primary media of the contagion, and the parasite as the secondary—analogous, in fact, to the atmosphere, simply a carrier, as it were.

Huter, Oertel and Eberth, and many others, on the other hand, present plausible grounds for concluding that the morbific agency of bacteria in disease is real and not imaginary. More or less confusion is the natural outgrowth of this controversy, and "outsiders" may consistently satisfy themselves with neutral ground until the contest is decided. As the character of infection is still in the dark, and as it is the nature of the human mind to observe and formulize, a very safe ground may be taken that it is a something unknown that produces it, and is just as apt to be fluid as gas, or algæ as fungi.

On this basis, whatever be the termination of the controversy, we will be in accordance with the principles established, however.

We are inclined to favor the parasitic theory, and have no doubt that as the chemical processes of putrefaction and fermentation are produced through the agency of fungi, it will be conclusively demonstrated that the algæ are principally instrumental in producing septic conditions which characterize some of our most fatal diseases.

